# Supplementary material for: Functional genomic analysis identifies miRNA repertoire regulating C. elegans oocyte development
Source: Nat Commun. 2018 Dec 14;9:5318. doi: 10.1038/s41467-018-07791-w (PMC6294007; doi:10.1038/s41467-018-07791-w)
Supplement: Supplementary file 1 — Supplementary Information [file 41467_2018_7791_MOESM1_ESM.pdf]

Functional Genomic Analysis Identifies miRNA Repertoire Regulating *C. elegans* Oocyte Development

Minogue et al.,

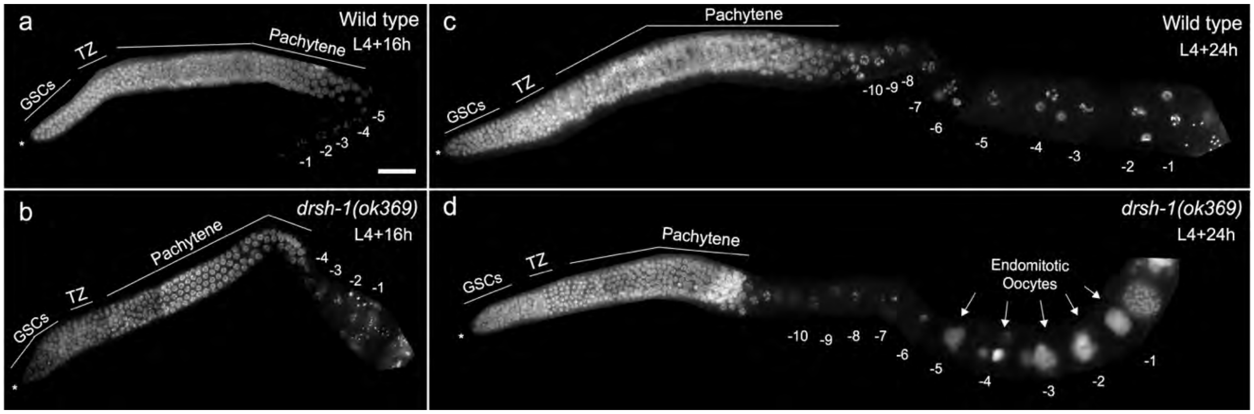

Supplementary Figure 1: *drsh-1(ok369)* mutants display endomitotic oocytes at 24 hours past the L4 stage.

Dissected gonads stained with DAPI (DNA, white) and oriented from left to right with oocytes on the right. * marks the distal tip cell. Scale bar: 20µM. (a) Wild type germlines at 16 hours past L4 stage of development exhibit no oogenic defects. (b) *drsh-1(ok369)* germlines at 16 hours past L4 stage of development reveal pachytene progression defects, and linear row or morphologically wild type appearing oocytes. (c) Wild type germlines at 24 hours past the L4 stage of development display morphological normal oocytes arrested in diakinesis. (d) *drsh-1(ok369)* germlines at 24 hours past the L4 stage of development exhibit multiple endomitotic oocytes (arrows).

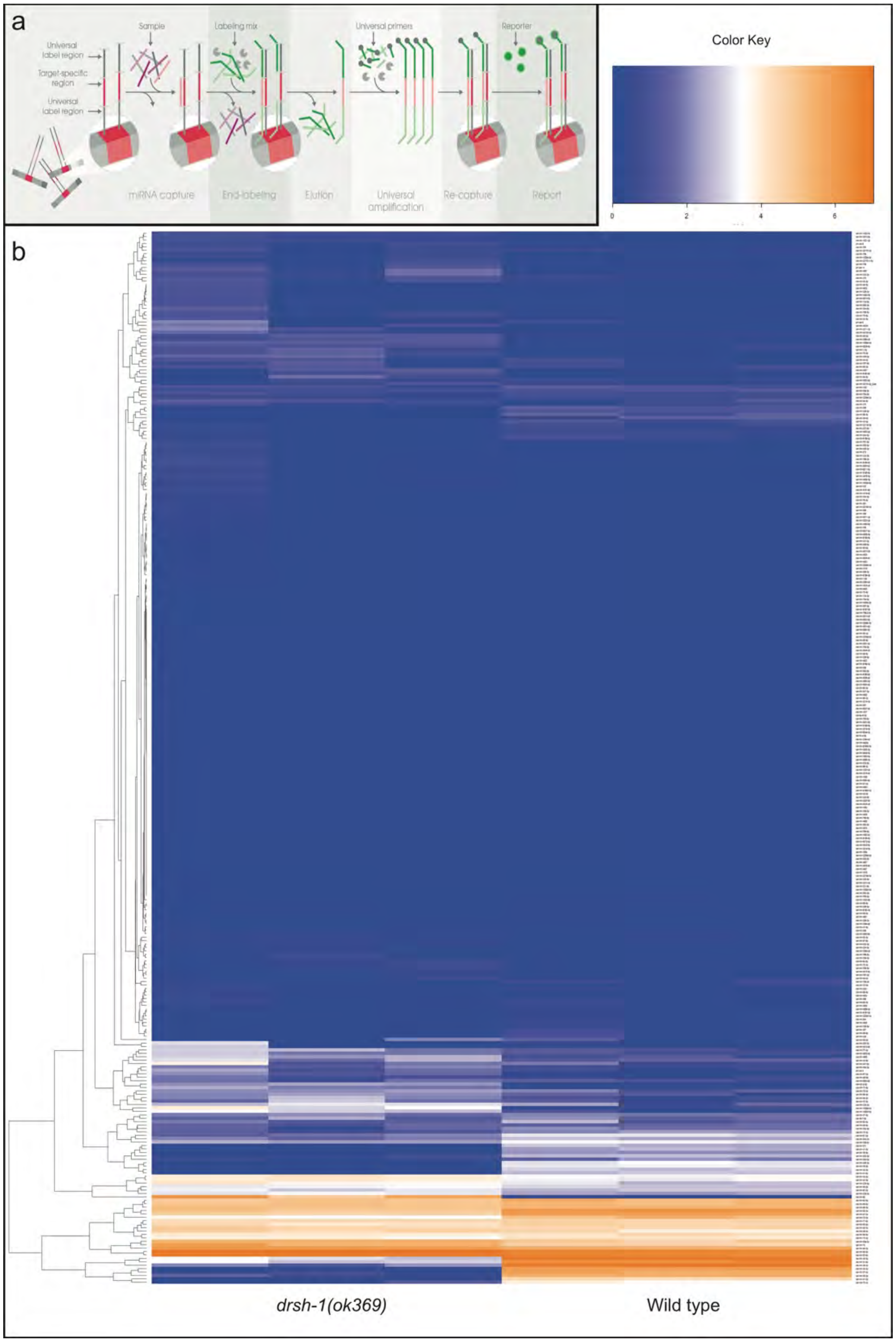

Supplementary Figure 2: FirePlex® analysis on wild type and *drsh-1(ok369)* dissected gonads for 307 mature *C. elegans* miRNAs.

(a) Schematic of FirePlex® miRNA profiling method. (b) Heat map analysis of the miRNAs identified from wild type and *drsh-1(ok369)* dissected germlines. Values / colors were assigned to reflect the mean fluorescent intensity values (MFI). Deep blue = low miRNA accumulation. Bright orange = high miRNA accumulation.

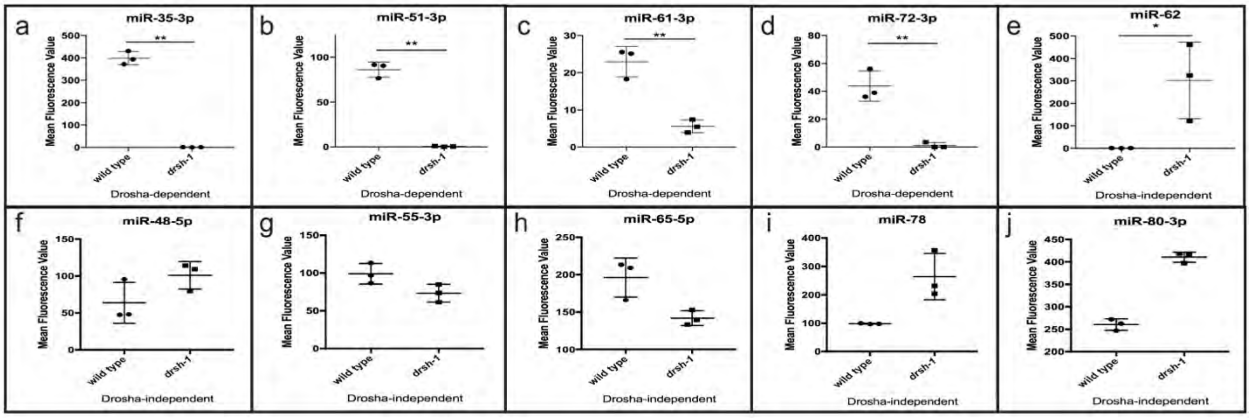

Supplementary Figure 3: Germline-expressed miRNAs are Drosha-dependent and independent.

Differential expression determined by a two-tailed Student's T-test with an adjusted P value using the Bonferroni's correction.

(a-d) Box plots of four representative *drosha*-dependent miRNAs mapping abundance of the miRNA in wild type and *drsh-1(ok369)*. (e-j) Box plots of six representative *drosha*-independent miRNAs mapping abundance of the miRNA in wild type and *drsh-1(ok369)*. * Denotes $P<0.05$ ** Denotes $P<0.01$. ±SD.

miRNA tested		Expression	Expression Pattern	FirePlex® Detected	Phenotype
***drosha*-dependent from FirePlex® analysis**	miR-35-3p	positive	Pattern 1	x	pachytene progression, fewer oocytes
	miR-36-3p	positive	Pattern 1	x	pachytene progression, fewer oocytes
	miR-37-3p	positive	Pattern 1	x	pachytene progression, fewer oocytes
	miR-38-5p	positive	Pattern 1		pachytene progression, fewer oocytes
	miR-39-3p	positive	Pattern 1	x	pachytene progression, fewer oocytes
	miR-40-3p	positive	Pattern 1	x	pachytene progression, fewer oocytes
	miR-61-3p	positive	Pattern 1	x	pachytene progression, fewer oocytes
	miR-51-5p	positive	Pattern 2	x	fewer oocytes
	miR-72-3p	positive	Pattern 2	x	fewer oocytes
	miR-72-5p	positive	Pattern 2	x	fewer oocytes
	miR-229-5p	positive	Pattern 3	x	N/A
	miR-51-3p	negative	N/A	x	fewer oocytes
***drosha*-independent from FirePlex® analysis**	miR-52-5p	positive	Pattern 2	x	N/A
	miR-54-3p	positive	Pattern 2	x	early nucleolar breakdown, fewer oocytes
	miR-65-5p	positive	Pattern 2	x	N/A
	miR-78	positive	Pattern 2	x	N/A
	miR-55-3p	positive	Pattern 3	x	early nucleolar breakdown, fewer oocytes
	miR-58a-3p	positive	Pattern 3	x	N/A
	miR-71-5p	positive	Pattern 3	x	N/A
	miR-73-3p	positive	Pattern 3	x	N/A
	miR-82-3p	positive	Pattern 3	x	N/A
	miR-80-3p	positive	Pattern 4	x	no phenotype
	miR-81-3p	positive	Pattern 4	x	N/A
	miR-53-5p	positive	Pattern 4	x	N/A
	miR-64-5p	negative	N/A	x	N/A
	miR-66-5p	negative	N/A	x	N/A
	miR-44-3p	negative	N/A	x	N/A
	miR-56-3p	negative	N/A	x	early nucleolar breakdown, fewer oocytes
	miR-48-5p	negative	N/A	x	N/A
**Not detected as germline-expressed from either genomic method**	miR-80-5p	negative	N/A		no phenotype
	miR-228-5p	negative	N/A		N/A
	miR-238-3p	negative	N/A		N/A
	miR-244-5p	negative	N/A		N/A
	miR-41-3p	negative	N/A		pachytene progression, fewer oocytes
	miR-41-5p	negative	N/A		pachytene progression, fewer oocytes

Supplementary Figure 4: *In situ* hybridization analysis reveals spatial expression domains for germline miRNAs.

A table summarizing the miRNAs that were tested through *in situ* hybridization.
Column 1: *drosha*-dependent or independent classes.
Column 2: Positive or Negative expression of the miRNA via *in situ* analysis in the dissected germline.
Column 3: Patterns defined by regions of the germline that the miRNAs localize to.
Column 4: Identified by the FirePlex® method as germline expressed.
Column 5: Germline phenotypes of miRNA mutants.

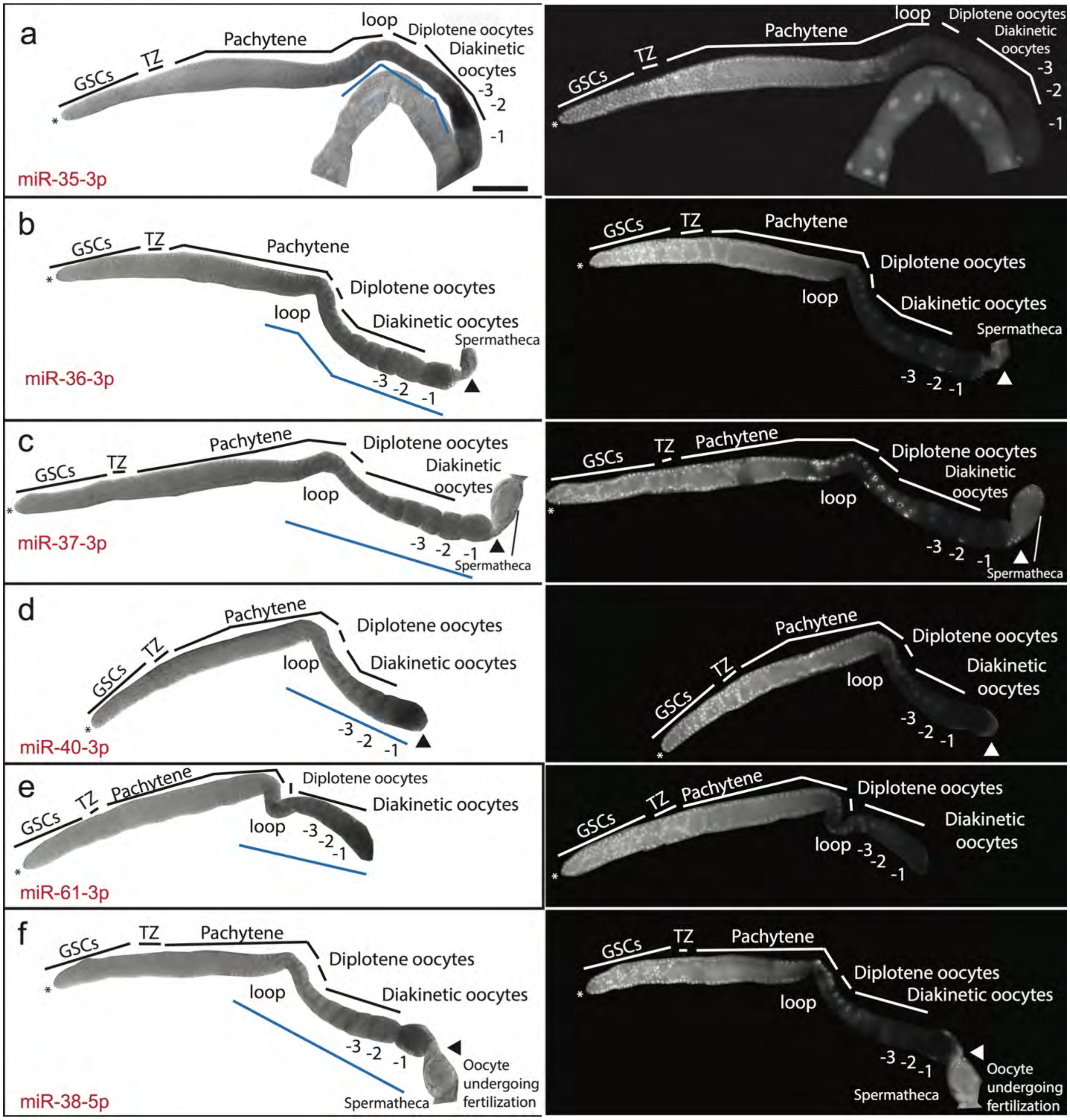

Supplementary Figure 5: Spatial expression for Pattern 1 miRNAs.
Dissected gonads oriented left to right, with oocytes to the right. * marks the distal tip cell. Bright field microscopy (left). DAPI (DNA, right) marks germ cell chromosomal stages. The blue line indicates regions of the germline with positive expression for the probe. Scale bar: 20μM (a) miR-35-3p. (b) miR-36-3p. (c) miR-37-3p. (d) miR-40-3p. (e) miR-61-3p. (f) miR-38-5p. Arrowhead marks the sperm.

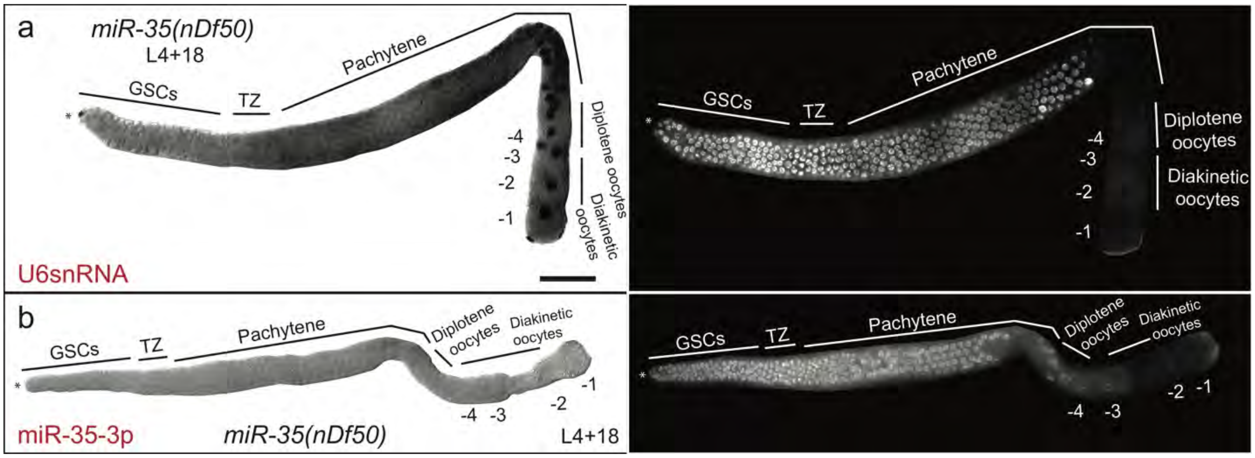

Supplementary Figure 6: miRNA *in situ* hybridization detects germline miRNAs with specificity.

Dissected gonads oriented left to right, with oocytes to the right. * marks the distal tip cell. Bright field microscopy (left). DAPI (DNA, right) marks germ cell nuclear stages. Scale bar: 20μM (a) The U6 small nucleolar RNA is expressed in all the germ cell nuclei, somatic distal tip cell (*) and sheath cell nuclei, in the *mir-35-41(nDf50)* germlines. (b) miR-35-3p is undetectable in the *mir-35-41*(*nDf50*) germlines.

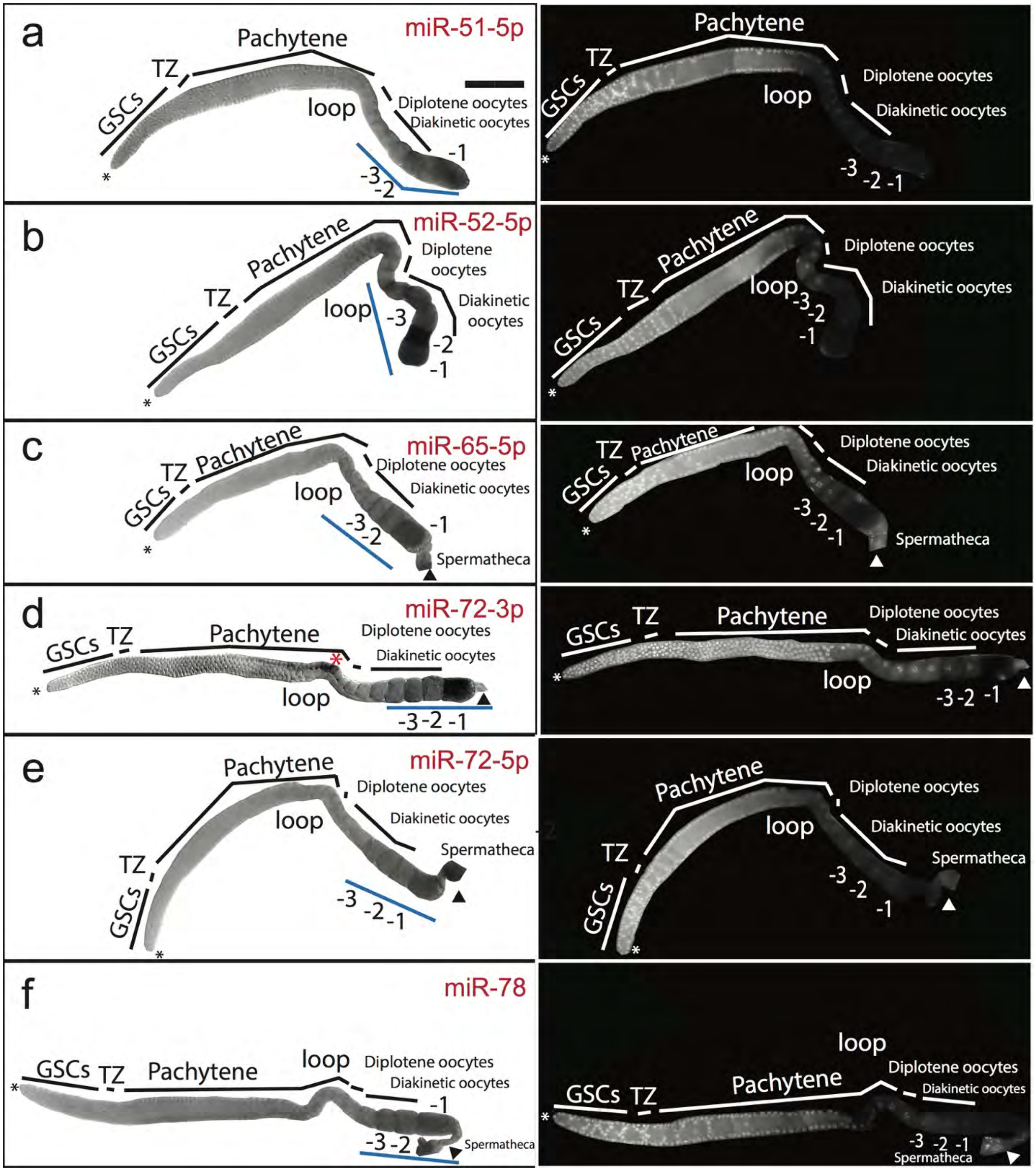

Supplementary Figure 7: Spatial expression of Pattern 2 miRNAs.
Dissected gonads oriented left to right, with oocytes to the right. * marks the distal tip cell. Bright field microscopy (left). DAPI (DNA, right) marks germ cell nuclear stages. The blue line indicates regions of the germline with positive expression for the probe. Scale bar: 20μM (a) miR-51-5p. (b) miR-52-5p. (c) miR-65-5p. (d) miR-72-3p. The red asterisk marks signal artifact. (e) miR-72-5p. (f) miR-78. Arrowhead marks the sperm.

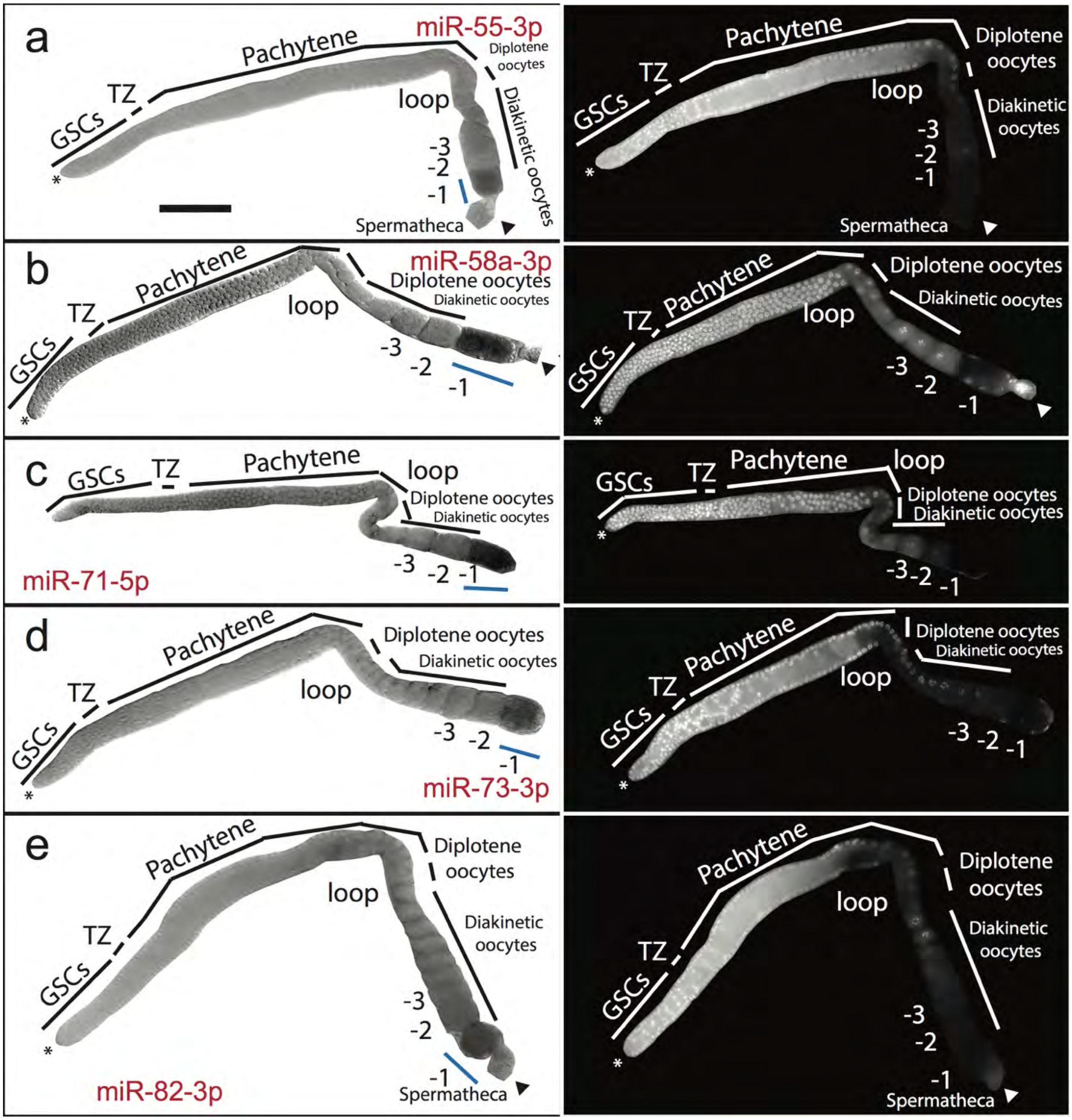

Supplementary Figure 8: Spatial expression of Pattern 3 miRNAs.

Dissected gonads oriented left to right, with oocytes to the right. * marks the distal tip cell. Bright field microscopy (left). DAPI (DNA, right) highlights germ cell nuclear stages. The blue line indicates regions of the germline with positive expression for the probe. Scale bar: 20µM (a) miR-55-3p. (b) miR-58a-3p. (c) miR-71-5p. (d) miR-73-3p. (e) miR-82-3p. Arrowhead marks the sperm.

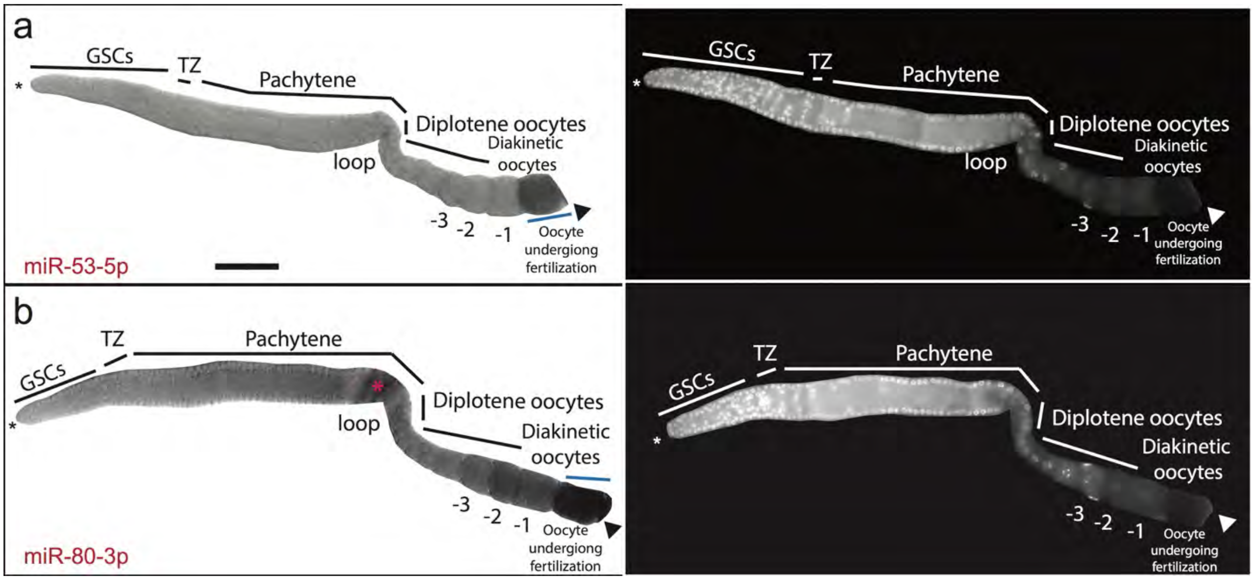

Supplementary Figure 9: Spatial expression of Pattern 4 miRNAs.
Dissected gonads oriented left to right, with oocytes to the right. * marks the distal tip cell. Bright field microscopy (left). DAPI (DNA, right) to mark germ cell nuclear stages. The blue line indicates regions of the germline with positive expression for the probe. Scale bar: 20μM (a) miR-53-5p. (b) miR-80-3p. Arrowhead marks the sperm.

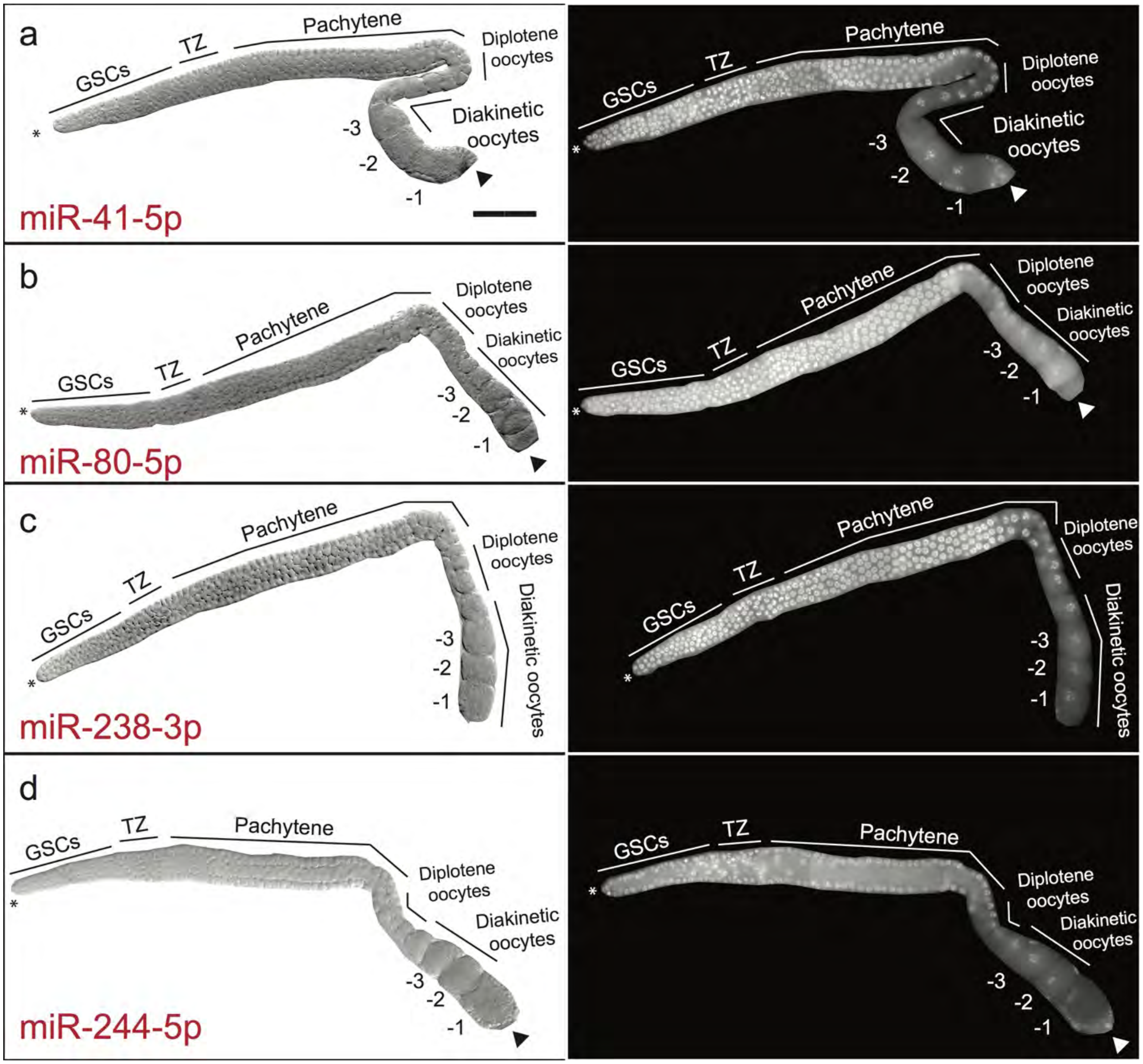

Supplementary Figure 10: *In situ* hybridization of miRNAs not identified by Fireplex® analysis as germline expressed.

Dissected gonads oriented left to right, with oocytes to the right. * marks the distal tip cell. Bright field microscopy (left) reveals lack of *in situ* expression. DAPI (DNA, right) marks germ cell nuclear stages. Scale bar: 20µM**.** Representative somatic miRNAs are shown. (a) *miR-41-5p.* (b) miR-80-5p*.* (c) miR-238-3p. (d) miR-244-5p. Arrowhead marks the sperm

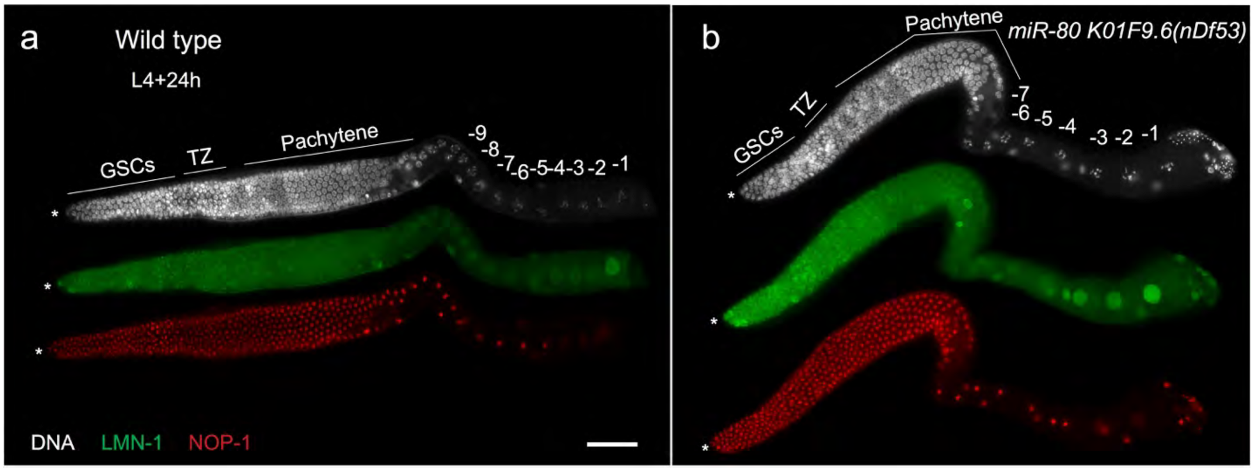

Supplementary Figure 11: *mir-80K01F9.6(nDf53)* mutant germlines do not display oogenic defects.

Dissected gonads stained with DAPI to visualize nuclear morphology (DNA, white), NOP-1 (nucleolus, red), and LMN-1 (Lamin marks nuclear membrane, green) oriented from left to right with oocytes on the right. * marks the distal tip cell. Scale bar: 20µM. (a) Wild type germlines exhibit 9-11 oocytes with no oocyte defects. (b) *mir-80K01F9.6(nDf53)* mutant germlines display wild type germline morphology and number of oocytes.

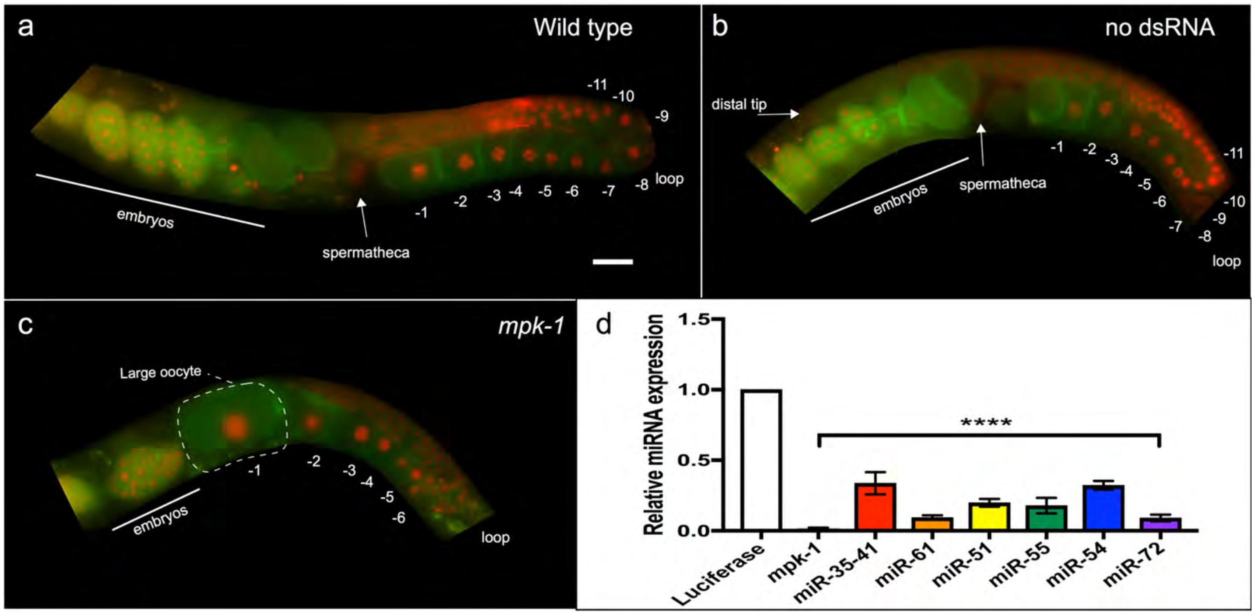

Supplementary Figure 12: Positive and negative controls used in the soaking RNAi experiment.

The photograph displays live images of germlines from whole animals bearing membrane GFP (green) and Histone 2B mCherry (red). The animal is oriented with dorsal (top) ventral (bottom) polarity. Scale bar: 20µM. (a) Wild type untreated animals (on plates) do not display any germline phenotypes. (b) Wild type untreated animals (no dsRNA) soaked in RNAi buffer do not display any germline phenotypes. (c) *mpk-1* RNAi treatment on soaking results in large and disorganized oocytes. (d) TaqMan™ analysis of relative miRNA knockdown from each RNAi treatment group (Figure 4) shows a significant knockdown. TaqMan™ assay was repeated for each of the three RNAi soaking replicates, and statistical significance was calculated by a one tailed ANOVA with Bonferroni correction. ****$P<0.0001$. ±SD.

	Minogue et al., Germline-enriched	Minogue et al., Drosha-dependent	Brown et al., Alg-5 IP
cel-mir-35-3p	X	X	
cel-mir-36-3p	X	X	X
cel-mir-37-3p	X	X	X
cel-mir-39-3p	X	X	X
cel-mir-40-3p	X	X	X
cel-mir-51-3p	X	X	X
cel-mir-51-5p	X	X	X
cel-mir-61-3p	X	X	X
cel-mir-72-3p	X	X	X
cel-mir-72-5p	X	X	X
cel-mir-229-5p	X	X	
cel-mir-44-3p	X		
cel-mir-45-3p	X		
cel-mir-48-5p	X		
cel-mir-52-5p	X		
cel-mir-53-5p	X		
cel-mir-54-3p	X		
cel-mir-55-3p	X		
cel-mir-56-3p	X		
cel-mir-58a-3p	X		
cel-mir-64-5p	X		
cel-mir-65-5p	X		
cel-mir-66-5p	X		
cel-mir-71-5p	X		
cel-mir-73-3p	X		
cel-mir-78	X		
cel-mir-80-3p	X		
cel-mir-81-3p	X		
cel-mir-82-3p	X		
cel-mir-35-5p			X
cel-mir-36-5p			X
cel-mir-39-5p			X
cel-mir-41-3p			X
cel-mir-58b-3p			X
cel-mir-61-5p			X
cel-mir-250-3p			X
cel-mir-250-5p			X
cel-mir-253-3p			X
cel-mir-1829a-3p			X
cel-mir-1829a-5p			X
cel-mir-1829b			X
cel-mir-1829c			X
cel-mir-4812-3p			X
cel-mir-4813-3p			X

Supplementary Figure 13: Oocyte expressed miRNAs identified in this study, and their overlap with ALG-5 enriched miRNAs.

X indicates that the miRNA was identified in the study.

Gray box: miRNAs that were not identified in the study.

Yellow box: miRNAs were positively identified in the study.

drosha-dependent oocyte expressed miRNAs from this study were also identified to be ALG-5 enriched miRNAs (column 3). *drosha*-independent miRNAs (this study) were not enriched in the ALG-5 enriched populations[1].

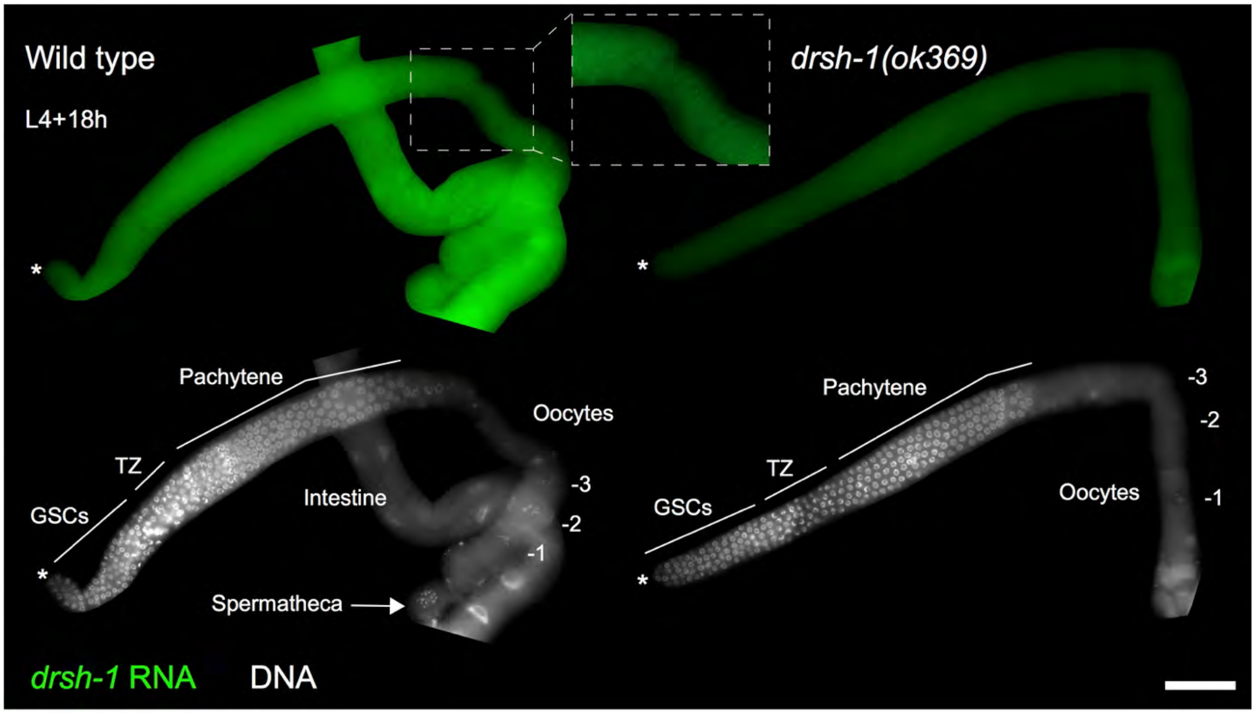

Supplementary Figure 14: *drsh-1* mRNA is expressed throughout the germline and the intestine in wild type, and reduced in *drsh-1*(*ok369*) allele.

Hairpin chain reaction based *in situ* analysis reveals *drsh-1* mRNA to be expressed throughout the germline (left, wild type) and the intestine. *drsh-1* mRNA is reduced in the *drsh-1*(*ok369*) mutant germlines.

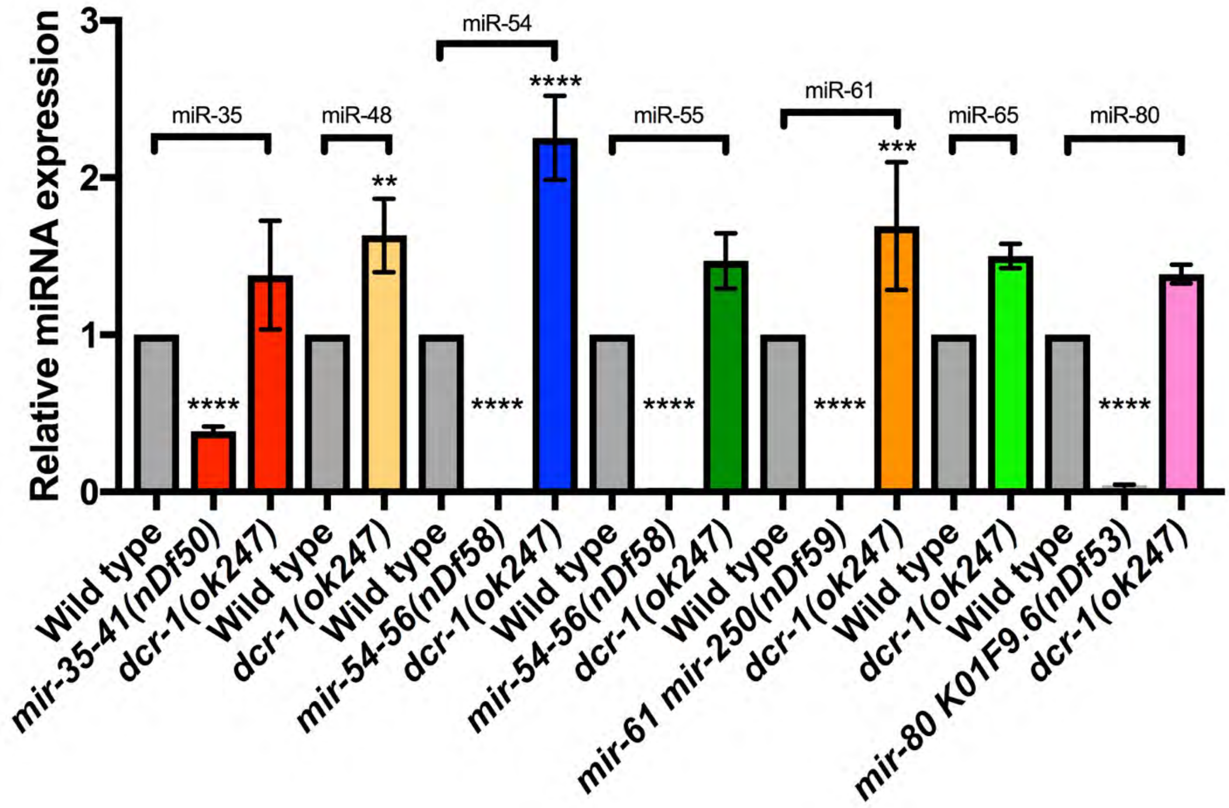

Supplementary Figure 15: *drosha*-independent miRNAs perdure in the *dcr-1*(*ok247*) mutant animals.

TaqMan™ analysis on whole animals (wild type, respective miRNA mutants, and *dcr-1*(*ok247*) homozygous allele) at 18 hours past the mid-L4 stage of development reveals that the *drsh-1* independent miRNAs perdure in the *dcr-1*(*ok247*) allele. This is consistent with our previous analysis conducted with next generation sequencing on *dcr-1*(*ok247*) mutant animals[2].

miRNA		Seed Sequence:	Genomic Localization:	Part of a cluster?
drosha -dependent from FirePlex® analysis	miR-35-3p	caccggg	intronic, within intron 2 of Y62F5A.9	yes, miR-35-41
	miR-36-3p	caccggg	intronic, within intron 2 of Y62F5A.9	yes, miR-35-41
	miR-37-3p	caccggg	intronic, within intron 2 of Y62F5A.9	yes, miR-35-41
	miR-39-3p	caccggg	intronic, within intron 2 of Y62F5A.9	yes, miR-35-41
	miR-40-3p	caccggg	intronic, within intron 2 of Y62F5A.9	yes, miR-35-41
	miR-51-3p	auggaag	intergenic, between alh-3 and F36H1.17	yes, miR-51 and miR-53
	miR-51-5p	acccgua	intergenic, between alh-3 and F36H1.17	yes, miR-51 and miR-53
	miR-61-3p	gacuaga	intergenic, between sel-11 and F55A11.4	yes, miR-61 and miR-250
	miR-72-3p	gcuucgc	intergenic, between vab-19 and pqn-42	no
	miR-72-5p	ggcaaga	intergenic, between vab-19 and pqn-42	no
	miR-229-5p	augacac	intronic, within intron 11 of gcn-1	yes, miR-64-66 and miR-229
drosha -independent from FirePlex® analysis	miR-44-3p	gacuaga	intergenic, between linc-47 and and ZK930.2	yes, miR-42-45
	miR-45-3p	gacuaga	intergenic, between top-2 and ZK930.5	yes, miR-42-45
	miR-48-5p	gagguag	intergenic, between F56A12.6 and F56A12.8	yes, miR-48 and miR-241
	miR-52-5p	acccgua	intergenic, between Y37A1B.330 and 21ur-2841	no
	miR-53-5p	acccgua	intergenic, between F36H1.17 and hrg-5	yes, miR-51 and miR-53
	miR-54-3p	acccgua	intronic, within intron 1 of F09A5.3	yes, miR-54-56
	miR-55-3p	acccgua	intronic, within intron 1 of F09A5.3	yes, miR-54-56
	miR-56-3p	acccgua	intronic, within intron 1 of F09A5.3	yes, miR-54-56
	miR-58a-3p	gagaucg	intronic, within intron 8 of Y67D8A.2	no
	miR-62	gauaugu	mirtron, within intron 3 of ugt-50	no
	miR-64-5p	augacac	intronic, within intron 11 of gcn-1	yes, miR-64-66 and miR-229
	miR-65-5p	augacac	intronic, within intron 11 of gcn-1	yes, miR-64-66 and miR-229
	miR-66-5p	augacac	intronic, within intron 11 of gcn-1	yes, miR-64-66 and miR-229
	miR-71-5p	gaaagac	intronic, within intron 19 of ppfr-1	yes, miR-2 and miR-71
	miR-73-3p	ggcaaga	intergenic, between T24D8.11 and T24D8.14	yes, miR-73-75
	miR-78	ggaggcc	intergenic, between Y40H7A.3 and Y40H7A.4	no
	miR-80-3p	gagauca	intergenic, between plg-1 and K01F9.2	yes, miR-80, miR-90, and miR-238
	miR-81-3p	gagauca	intronic, within intron 5 of T07D1.2	yes, miR-81-82
	miR-82-3p	gagauca	intronic, within intron 5 of T07D1.2	yes, miR-81-82

Supplementary Figure 16: Genomic localization of *drosha*-dependent and independent miRNAs

Germline enriched miRNAs identified in this study localize to both intronic and intergenic regions of the genome. miR-62 (orange) is an annotated mirtron.

on, also identified to be Drosha independent in this study.

Column 1, gray shading: Drosha dependent miRNAs identified in this study.

Column 1, green shading: Drosha independent miRNAs identified in this study.

Column 2, shared miRNA seed sequences among the oocyte-expressed miRNAs identified by similar shading.

Column 3, genomic location of each of the oocyte-expressed miRNAs. Gray shading marks miRNAs that occur in intronic regions. Orange shading marks mirtron, miR-62, previously identified[3].

Column 4, indicates whether the miRNA displayed is part of a larger cluster.

SUPPLEMENTARY REFERENCES

1 Brown, K. C., Svendsen, J. M., Tucci, R. M., Montgomery, B. E. & Montgomery, T. A. ALG-5 is a miRNA-associated Argonaute required for proper developmental timing in the Caenorhabditis elegans germline. *Nucleic Acids Res* **45**, 9093-9107, doi:10.1093/nar/gkx536 (2017).

2 Drake, M. *et al.* A requirement for ERK-dependent Dicer phosphorylation in coordinating oocyte-to-embryo transition in C. elegans. *Dev Cell* **31**, 614-628, doi:10.1016/j.devcel.2014.11.004 (2014).

3 Ruby, J. G., Jan, C. H. & Bartel, D. P. Intronic microRNA precursors that bypass Drosha processing. *Nature* **448**, 83-86, doi:10.1038/nature05983 (2007).
