# Supplementary material for: Functional genomic analysis identifies miRNA repertoire regulating C. elegans oocyte development
Source: Nat Commun. 2018 Dec 14;9:5318. doi: 10.1038/s41467-018-07791-w (PMC6294007; doi:10.1038/s41467-018-07791-w)
Supplement: Supplementary file 3 — Description of Additional Supplementary Information [file 41467_2018_7791_MOESM3_ESM.pdf]

Description of Additional Supplementary Files

File Name: Supplementary Data 1
Description: **FirePlex® analysis on dissected gonads from wild type and *drosha(0)* mutants for 307 miRNAs**. Each individual biological triplicate experiment conducted by FirePlex® platform is displayed per row for wild type or *drosha(0)* dissected gonads. Each miRNA is represented in the columns. The values represent mean fluorescence intensity of the miRNA probe when compared to background. Light blue shading represents germline expressed miRNAs. Yellow shading represents known mirtons.
